# The Prevalence and Influence Factors of Inter-Ankle Systolic Blood Pressure Difference in Community Population

**DOI:** 10.1371/journal.pone.0070777

**Published:** 2013-08-27

**Authors:** Zhihong Zhang, Jianyong Ma, Xuehua Tao, Yueying Zhou, Xin Liu, Hai Su

**Affiliations:** 1 Research Institute of Cardiovascular Diseases and Department of Cardiology, Second Affiliated Hospital of Nanchang University, Nanchang, Jiangxi, People's Republic of China; 2 Statistic Teaching Group, Fuzhou Medical College of Nanchang University, Fuzhou, Jiangxi, China; 3 Department of Medicine, Sixth Hospital of Nanchang City, Nanchang, Jiangxi, People's Republic of China; 4 Department of Medicine, Guizhou Hospital of Fushan City, Fushan, Guangdong, People's Republic of China; University of Sao Paulo Medical School, Brazil

## Abstract

**Aim:**

The aim of this study was to investigate the prevalence of interankle systolic blood pressure difference (sIAND) and its influencing factors in community population.

**Methods:**

This study included 2849 (65.1±9.4 y) subjects. Blood pressure (BPs) of four limbs was simultaneously measured with 4 electronic sphygmomanometers after 10 min rest in supine position. The difference of systolic BP (SBP) between two ankles was calculated as DETASBP. The criterion for abnormal sIAND was ≥10 mmHg of absolute DeltaSBP, in which the criterion for 1o sIAND was 10–19 mmHg and for 2o sIAND was ≥20 mmHg. Age, gender, smoking, hypertension, family histories of hypertension and diabetes were recorded. Fasting blood glucose and lipids, circumference of hip and waist, and body mass index (BMI) were measured.

**Results:**

The SBP was higher in the right ankle than in the left ankle (158.9±21.8 vs 157.3±21.6 mmHg, P<0.05) and mean DeltaSBP was 6.08±6.26 mmHg. Similar difference was found in both genders. The prevalence of abnormal was 18.5%, in which, the prevalence 1o sIAND was 15.3% and that of 2o sIAND was 3.1%. Multivariate regression analysis showed that age, waist circumference and blood glucose level were the positive factors for DeltaSBP. The normal upper limit for DeltaSBP was 16.7 mmHg in this population, the prevalence of sIAND by≥16 mmHg was 5.8%.

**Conclusion:**

Aging, hypertension, obesity and abnormal glucose metabolism are positive factors for inter-ankle SBP difference.

## Introduction

The guidelines for hypertension and blood pressure measurement point out that for the first blood pressure (BP) taking, two arms should be measured to evaluate interarm BP difference (IAD) [1]–[3].

Increasing data show that IAD is not only a sign for diagnosing subcravian and brachial artery stenosis, left ventricular hypertrophy, but also a predictor of adverse cardiovascular outcomes [4]–[6].

In recent years, some studies suggested that simultaneously measuring BP of four limbs can provide more valuable information, for example, an ankle-brachial index (ABI). A low ABI (<0.9) is an important sign for intermittent claudication and other peripheral vascular disease. Meanwhile, ABI is also a useful predictor for cardio-cerebrovascular diseases [7], [8]. Recently, as ankle BP can be easily measured, it is used more often in clinical practice. Some studies have demonstrated that ankle BP is a useful BP index. A study showed that the leg BP in orthostatic posture is associated with left ventricular mass in normotensive subjects [9]. Furthermore, increased ankle BP is a marker of arterial stiffness or subclinical atherosclerosis, and an independent risk for future dementia, cardio-cerebrovascular morbidity and mortality [10],[11].

Like arms, two ankles in some patients may show significantly different BP, i.e., interankle BP difference (IAND). Recently, some studies showed that IAND could predict the risk for overall and cardio-cerebrovascular events (12,13). Because IAND was not widely studied, in the previous studies various diagnostic criteria were used: mean values, or quartile, or ≥10 mm Hg, or ≥15 mm Hg [12],[13].

One reason for this confusion phenomenon is no data about the prevalence of IAND in community population. This study was to investigate the prevalence of IAND in community population and to evaluate the possible influencing factors for IAND.

## Methods

### Study Participants and data Collection

This study enrolled 3311 adult community residents of two different communities during Sept–Dec of 2011, one in Nanchang City of Jiangxi province and the other in Shunde City of Guandong province. The information on age, sex, smoking (more than half year), family history of hypertension, histories of hypertension and diabetes were obtained with questionnaires and medical records. Waist and hip circumference, body mass index (BMI), fasting blood glucose, total cholesterol (TC), triglyceride (TG), high and low density lipoprotein cholesterol (HDL-C and LDL-C) were measured. Ratios of LDL-C/HDL-C and LDL-C/TC were calculated. The participants under antihypertensive therapy or with BP of 140/90 mmHg or more at investigation were diagnosed as hypertensive patients. Subjects with arrhythmias and known intermittent claudication were excluded. Finally, 2849 subjects of ≥40 years (854 males and 1995 females, 40–108 years old, mean age 65.0±9.3 y) with complete measurement data were included.

### BP measurements and parameters

This study focused on systolic IAND (sIAND). After 10-minute supine rest, supine BP of four limbs were measured simultaneously using 4 electronic sphygmomanometers (Omron HEM7101) for three times with a 2-minute interval. Their averages were used to calculate absolute interankle SBP difference (DETA BP). When DETASBP was 10–19 mm Hg class 1 sIAND (1o s IAND) and DETASBP was ≥20 mmHg class 2 sIAND (2o sIAND) were diagnosed [14],[15]. The higher SBP of two ankles was used as individual ankle SBP value to calculate ratio of DeltaSBP/SBP (%).

The higher SBP of two arms and the lower SBP of two ankles were used to calculate sABI, and sABI of <0.9 was considered as abnormal.

The proposal and the consent procedures of this study were approved by the Ethic Committees of the Second Affiliated Hospital of Nanchang University, and of the Sixth Hospital of Nanchang city, and of Guizhou Hospital of Fushan city. All patients provided verbal informed consent for BP measurement of four limbs as this measurement is a non-invasive clinical examination.

### Statistical analysis

All data are presented as Mean ± Standard Deviation (Mean±SD). The SPSS10.0 statistical package (SPSS Company, Chicago, Illinois, USA) was used for Student's t-test and Chi-square test in this study. Linear correlation analysis was used for the relationship between DeltaSBP or DeltaSBP/SBP and other variables.

In multivariate analysis, the independent variables were SBP and SBP/SBP, and the dependent variables were age, gender (male 1, female 0), BMI (kg/m2), waist circumference, hip circumference, smoking (yes 1, no 0), history of hypertension (yes 1, no 0), diabetes (yes 1, no 0), coronary heart disease (yes 1, no 0), TG, TC, LDL-C/HDL-C, LDL-C/TC and blood glucose levels. The definition of the normal upper limit of DETASBP *was* the reference value range of 95% on<mean+1.64SD. Statistical significance was defined as P<0.05.

## Results

### The general information between two gender groups

The general information is shown in Table 1. The mean age and smoking rate were higher in the male group than those in the female group (Table 1).

**Table 1:** Comparison of general information of two gender groups.

Gender (n)	Age(y)	BMI(kg/m2)	Smokingn (%)	History of HHn (%)	History of DMn (%)	History of CHDn (%)
M(854)	66.6±9.1	23.4±3.2	260(30.4)	300(35.1)	118(13.8)	26(3.0)
F (1995)	64.5±9.5^a^	23.5±3.5	77(3.9)^a^	681(34.1)	234(11.7)	53(2.7)
Total (2849)	65.1±9.4	23.4±3.4	337(11.8)	981(34.4)	352(12.4)	79(2.8)

HH: hypertension, DM: diabetes, CHD: coronary heart disease.

a compared with females, p<0.05.

### Comparison of ankle SBP and sIAND prevalence between two gender groups

The right ankle SBP, either in males or in females, was about 1.7 mmHg higher than the left ankle SBP. Although the SBP was higher in males, DeltaSBP had no gender difference. In this population the normal upper limit for DeltaSBP was 16.7 mmHg.

Increased sIAND was found in 526 subjects (18.4%), of them, 437subjects (15.3%) were diagnosed 1o sIAND and 89 subjects (3.1%) 2o sIAND. The prevalence of 10 sIAND was significantly higher in males than in females, but the prevalence of 20 sIAND was similar between two gender groups (Table 2).

**Table 2:** Comparison of ankle SBP and sIAND prevalence between two gender groups.

	Right ankle(mm Hg)	Left ankle(mm Hg)	ΔSBP(mm Hg)	ΔSBP/SBP(%)	No-sIAND(n,%)	Abnormal sIAND(n,%)	1^0^ sIAND(n,%)	2^0^sIAND(n,%)
M (854)	162.9±21.5	161.2±20.8^b^	6.48±7.10	4.06±4.45	673(78.8)	181(21.2)	153(17.9)	28(3.3)
F (1995)	157.3±21.7^a^	155.6±21.7^a^ ^b^	5.92±5.86^a^	3.85±3.79	1650(82.7)^a^	345(17.3)^a^	284(14.2)^a^	61(3.1)
Total (2849)	158.9±21.8	157.3±21.6^b^	6.08±6.26	3.91±4.00	2323(81.5)	526(18.5)	437(15.3)	89(3.1)

a compared with males, p<0.05;

b compared with right ankle, p<0.05.

Linear correlation analysis showed that DeltaSBP and DeltaSBP/SBP were positively correlated with age, BMI, waist circumference, hip circumference, blood glucose and TC levels. Meanwhile, DETASBP and DETASBP/SBP were positively correlated with baseline SBP also (Table 3).

**Table 3:** The correlation coefficients of ΔSBP and ΔSBP/SBP with related factors.

Parameters	ΔSBP		ΔSBP/SBP	
	*r*	*P*	*r*	*P*
age	0.090	<0.001	0.062	0.001
BMI	0.081	<0.001	0.052	0.006
waist	0.105	<0.001	0.072	<0.001
Hip	0.078	<0.001	0.049	0.009
TC	0.047	0.012	0.044	0.019
glucose	0.092	<0.001	0.059	0.010
SBP	0.219	<0.001	0.096	<0.001
DBP	0.156	<0.001	0.087	<0.001
ΔDBP	0.230	<0.001	0.228	<0.001

### Multivariate regression analysis

Multivariate regression analysis showed that age, waist circumference, blood glucose levels and hypertension history were the positive factors for DeltaSBP. Meanwhile, age, waist circumference and blood glucose levels were the positive factors for DeltaSBP/SBP (Table 4).

**Table 4:** The results of multifactor regression analysis.

*y*	*x*	Unstandardized Coefficients		Standardized Coefficients	*t*	*P*	95% Confidence Interval for B	
		B	Std. Error	Beta			Lower Bound	Upper Bound
ΔSBP	(Constant)	−3.013	1.331	-	−2.265	0.024	−5.622	−0.404
	waist	0.055	0.013	0.079	4.130	0.000	0.029	0.081
	Age	0.047	0.013	0.071	3.713	0.000	0.022	0.072
	Glucose	0.266	0.076	0.066	3.490	0.000	0.117	0.416
	History of HH	0.549	0.256	0.042	2.144	0.032	0.047	1.051
ΔSBP/SBP	(Constant)	−0.193	0.754	-	−0.256	0.798	−1.672	1.285
	waist	0.024	0.008	0.059	3.081	0.002	0.009	0.038
	Glucose	0.129	0.044	0.055	2.915	0.004	0.042	0.217
	Age	0.021	0.007	0.054	2.857	0.004	0.006	0.035

### The relation between sIAND and sABI

**Figure 1:**
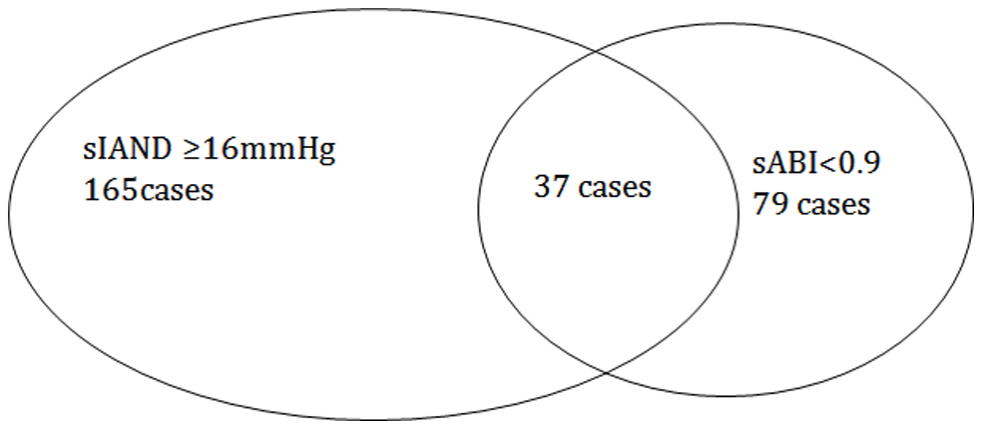
The relationship between increased sIAND and abnormal sABI.

The sABI was slightly higher in the female group than in the male group, but the prevalence of abnormal sABI was similar between two gender groups. A negative correlation was found between sIAND and sABI (R = −0.395, p<0.001) (Table 5). In this population, the prevalence of sIAND detected by≥16 mmHg was 5.8% (165cases), while, the prevalence of abnormal ABI(<0.9) was 2.8%. Only 37 of the 165 cases were included in the 79 cases with abnormal ABI. The overlapping subjects was 46 for the 526 sIAND cases detected by≥10 mmHg and 31 for the 89 sIAND cases by≥20 mmHg (Figure 1).

**Table 5:** Comparison of sABI between two gender groups.

Gender (n)	sABI(mean±SD)	sABI<0.9(%)
M(854)	1.12±0.09	58(2.9)
F (1995)	1.10±0.09^a^	21(2.5)
Total (2849)	1.11±0.09	79(2.8)

a Compared with males, p<0.05.

## Discussion

Traditionally, leg BP indicates the popliteal artery BP. Normally, the popliteal artery SBP is 20–40 mmHg higher than the brachial artery SBP. As ankle BP can be easily taken using electronic sphygmomanometer, it is used more often in clinical practice. Ankle BP is correlated with arm BP and the augmentation of central BP [15]. Elevated ankle BP was demonstrated as a risk for left ventricular hypertrophy and arterial stiffness, as well as a predictor for future dementia and cardio-cerebrovascular morbidity and mortality [9]–[11].

Intermittent claudication is a common disease caused by leg artery stenosis or occlusion. At present, palpation of dorsal artery of foot is often used to diagnose this disease. Because palpation method is insensitive, the diagnosis of intermittent claudication may be missed, especially in the early stage. On the basis of the value of systolic IAD for diagnosing subclavian or brachial artery stenosis [4],[5],[13],[16], sIAND may be considered as a useful for leg artery stenosis. In 210 hemodialysis patients, the sIAND of ≥15 mmHg was demonstrated a sign for peripheral vascular disease (11). Meanwhile, elevatedsIAND has been demonstrated a predictor for increased total mortality and cardiovascular mortality [11], [12]. Unfortunately, no unified diagnosis criterion was reported and accepted now [12],[17].

This phenomenon will limit the use of sIAND in clinical practice. Combining our results and the previous data, we may suggest ≥16.0 mmHg as a cut-off value for sIAND in adults aged 40 years and over. The present study demonstrated that age, waist circumference and blood glucose levels were positively BP-independent factors for increased sIAND, because they were positively correlated not only with DeltaSBP, but with DeltaSBP/SBP. Although hypertension history was positively associated with DeltaSBP, but not with DeltaSBP/SBP, it may be a SBP-dependent factor for sIAND. Meanwhile, the present study indicated that sIAND is independent of gender as both genders had similar DETASBPs and gender was not correlated with DeltaSBP and DeltaSBP/SBP. Even the prevalence of 10 sIAND was higher in males (17.9% vs14.2%), this gender difference for the prevalence of 10 sIAND may come from other factors existing in male group, such as higher mean age and smoking rate. Because aging, obesity and abnormal glucose metabolism are important risk factors of atherosclerosis, we consider that underlying mechanism of the increased DeltaSBP is leg artery atherosclerosis in middle-aged and elderly people.

The present study showed a negative relationship between sIAND and sABI. The reason for this relationship is clear, as increased sIAND and decreased sABI are related with leg artery stenosis. However, the clinical value of sIAND could not be replaced by sABI as they are dependent on the pathological condition of different limbs. If a patient has stenosis in both arms and in a leg, his or her sABI (as well as systolic interarm BP difference) may be “pseudomorphic normal”. In this case, only sIAND can provide valuable information. Meanwhile, IAD and ABI could not be correctly evaluated in some patients, for example, the renal inadequacy patients with an artificial arteriovenous fistula in one arm. Furthermore, this study showed that the overlapping rate between sIAND ans sABI was not so high, only 37 of 165 cases with sIAND by ≥16 mmHg were included into the 79 cases with abnormal sABI. Even in the 89 cases with 20 sIAND, only 31 cases with abnormal sABI. These results mean that sIAND or sABI has itself application area.

In addition, the prevalence of sABI was 2.8% on the higher SBP of two arms and the lower SBP of the two ankles. The prevalence of sABI was more lower, only 1.8% on the SBP from right side limbs and 1.4% from the left side limbs. Using the SBPs of four limbs to identify sABI may be more reasonable for better screening intermittent claudication.

### Clinical implication

Palpation of the leg artery is usually used to diagnose intermittent claudication in clinical treatment, but this method is insensitive. For correct diagnosis, artery imaging form computer tomography or catheterization is needed. Compared with the above-mentioned expensive or invasive examinations, evaluating sIAND is simpler and cheaper. So sIAND may be a useful index for screening asymptomatic intermittent claudication.

### Limitation

Although we suggest DETA BBP≥16 mmHg as a reference of cut-off value for sIAND in adults aged 40 years and over, its clinical value should be demonstrated by artery imaging evidence in the future.

## Conclusion

In a community population, 18.5% of subjects showed DETASBP of ≥10 mmHg, but only 5.8% had DETA SBP of ≥16 mmHg, a normal upper limit suggested by this study. Aging, obesity and abnormal glucose metabolism are SBP-independent positive factors for increased sIAND.
